# Acute alcohol consumption and motivation to reduce drinking among injured patients in a Swedish emergency department

**DOI:** 10.1186/1940-0640-7-S1-A34

**Published:** 2012-10-09

**Authors:** Anna Trinks, Karin Festin, Preben Bendtsen, Per Nilsen

**Affiliations:** 1 Department of Medical and Health Science, Division of Community Medicine, University of Linköping, Linköping, Sweden; 2 Department of Medicine and Health, Linköping University, Linköping, Sweden

## 

Injuries constitute a major public health problem. Millions of people are injured each year, and acute drinking is a well-known risk factor for injuries. Research suggests that acknowledgment of alcohol as a factor in an injury enhances willingness to change drinking behavior, possibly because the patient becomes aware of the negative consequences of their drinking. We investigated the prevalence of acute alcohol consumption among injured patients in Sweden and examined the factors associated with motivation to reduce alcohol consumption among these patients. All injured patients aged 18–69 years were given a card by a triage nurse with a request to answer alcohol-related questions on a touch-screen computer. Patients who completed the test received a printout containing personalized feedback on his or her drinking habits as calculated by the computer program from the patient’s answers. Fifteen percent of injured patients were categorized as acute drinkers, and of these, 64% reported that their injury was connected with alcohol. There were significant differences for sociodemographic and drinking characteristics between acute drinkers and nonacute drinkers. Acute drinkers were categorized as risky drinkers to a much higher extent than nonacute drinkers. Acute drinkers had considerably higher average weekly alcohol consumption and engaged far more frequently in heavy episodic drinking than nonacute drinkers. Acute drinkers were motivated to reduce their alcohol intake to a greater extent than nonacute drinkers: 51% were in the action, preparation, and contemplation stages compared with 19% of nonacute drinkers. Results indicate that acute drinkers had considerably more detrimental alcohol consumption than nonacute drinkers and were more motivated to reduce their drinking than nonacute drinkers.

